# Deep learning-based identification of aberrant anterior tibial artery on knee MRI: a brazilian multicenter study

**DOI:** 10.1007/s00256-026-05198-z

**Published:** 2026-03-23

**Authors:** Andre Yui Aihara, Cassiano Pereira de Barros, Daisy Terumi Kase, Ayumi Aihara, Eduardo Moreno Júdice de Mattos Farina, Adham do Amaral e Castro, Paulo de Tarso Kawakami Perez, Lucas Ribeiro de Medeiros, Leonardo Kazunori Tsuji, Ana Carolina de Lima Augusto, Andre Cesar Ozawa Rodrigues, Felipe Campos Kitamura, Nitamar Abdala

**Affiliations:** 1 Graduate Program in Medicine (Clinical Radiology), Imaging Diagnosis Department, Universidade Federal de São Paulo (UNIFESP), São Paulo, Brazil; 2 Division of Radiology, Delboni Medicina Diagnóstica, DASA, São Paulo, Brazil; 3 Faculdade de Ciências Médicas, Santa Casa de Misericórdia de São Paulo, São Paulo, Brazil; 4 Hospital Israelita Albert Einstein, São Paulo, Brazil; 5 Department of Radiology, Fleury Medicina E Saúde, São Paulo, Brazil; 6 Division of Musculoskeletal Radiology, Rede Dor, São Paulo, Brazil; 7 Eden, Palo Alto, CA USA; 8 Department of Evidence-Based Medicine, Universidade Federal de São Paulo (UNIFESP), São Paulo, Brazil; 9 Hapvida Notredame Intermédica, São Paulo, Brazil

**Keywords:** Aberrant anterior tibial artery, Deep learning, Knee MRI, Orthopedic surgery, Surgical planning

## Abstract

**Objective:**

To develop and validate a deep learning model for the detection of aberrant anterior tibial artery (AATA) on axial T2-weighted knee MRI, given the surgical relevance of unrecognized AATA and the lack of automated detection tools.

**Materials and methods:**

This retrospective study included 70,260 MRI images from 2315 examinations (1441 without AATA and 874 with AATA) collected after institutional review board approval. Musculoskeletal radiologists performed image-level annotations. Data were split at the patient level into training, validation, and internal test sets; an independent dataset from another institution served as an external test set. A convolutional neural network was implemented in Python and PyTorch. Model performance was assessed at the patient level.

**Results:**

At the slice level, the model achieved an F1-score of 0.838 on the internal test set. Patient-level classification using the validation-derived threshold (0.17) yielded F1-scores of 0.966 on the validation set, 0.979 on the internal test set, and 0.786 on the external test set. The area under the receiver operating characteristic curve for the external cohort was 0.97, indicating strong generalization despite a decrease in precision due to false positives.

**Conclusion:**

To our knowledge, this is the first study to apply artificial intelligence for automated detection of AATA on knee MRI. The proposed deep learning model performs this task with high sensitivity. Despite reduced precision in the external cohort, it demonstrates strong potential for enhancing preoperative risk assessment and surgical planning. Broader multicenter validation is warranted before clinical deployment.

**Supplementary Information:**

The online version contains supplementary material available at 10.1007/s00256-026-05198-z.

## Introduction

**Fig. 1:**
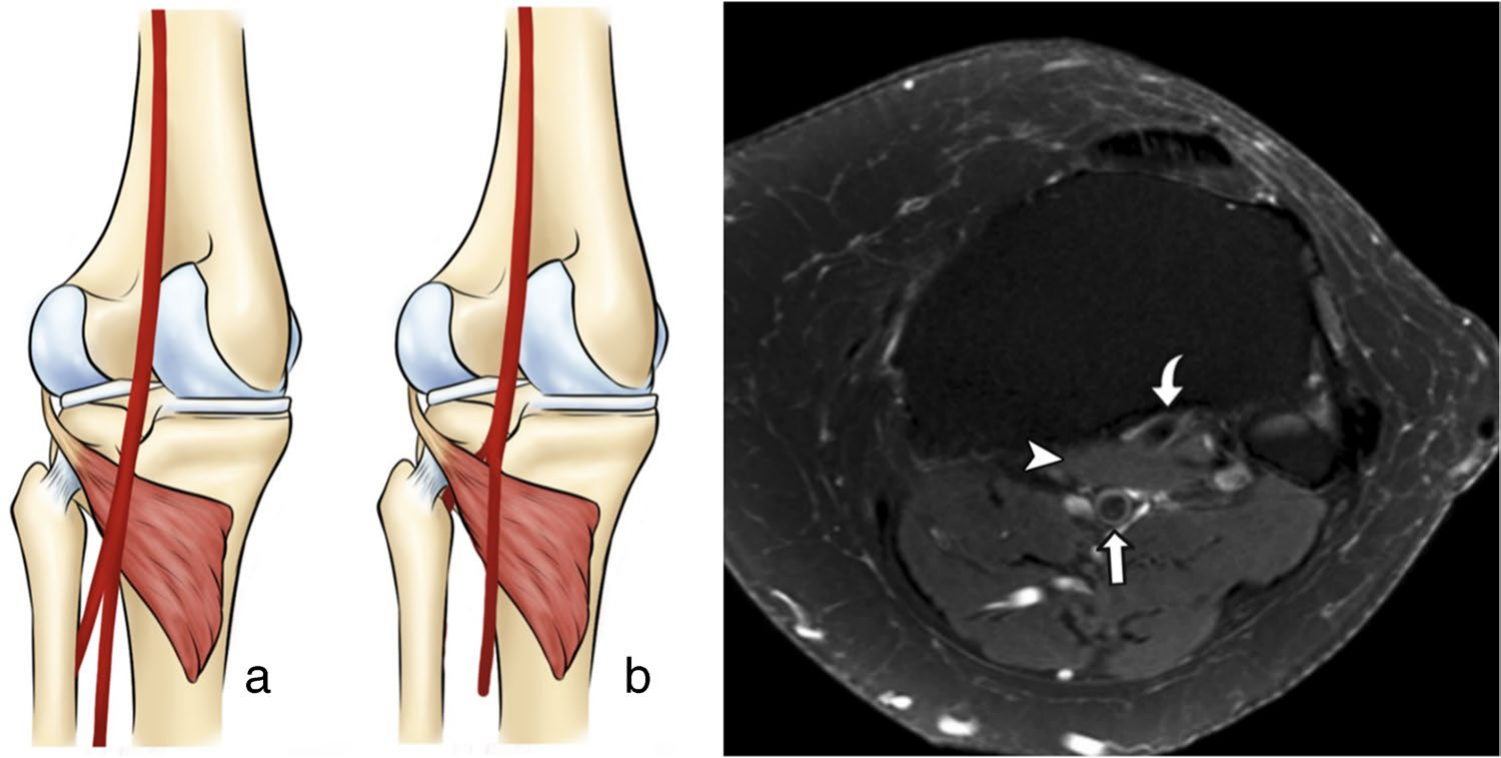
Anatomical illustration and MRI example of aberrant anterior tibial artery. On the left, a visual representation of normal (**a**) and aberrant (**b**) popliteal artery bifurcations, highlighting the course of the aberrant anterior tibial artery (curved arrow) in relation to the popliteus muscle (asterisk) and posterior tibial cortex. Normal anterior tibial artery (arrowhead) and tibioperoneal trunk (straight arrow) are also depicted. On the right (**c**), an axial T2-weighted fat suppressed knee MRI demonstrates the corresponding aberrant anterior tibial artery (curved arrow) coursing between the popliteus muscle (asterisk) and the posterior tibial cortex. Posterior tibioperoneal trunk (straight arrow) is also demonstrated

Injury to a popliteal fossa artery during orthopedic knee surgery is rare [1] but can have severe consequences, including pseudoaneurysm or severe bleeding [2, 3]. The risk of vascular trauma may increase in the presence of an aberrant anterior tibial artery (AATA), a variant in which the anterior tibial artery originates above the popliteus muscle and runs between the posterior tibial cortex and the ventral margin of the popliteus muscle (Fig. 1) [1, 4, 5]. The prevalence of this variation ranges from 0.4 to 6% [1, 3], depending on the diagnostic and imaging modality used. Due to its close proximity to surgical instrumentation, the AATA is particularly vulnerable during procedures such as high tibial osteotomies, where injury has already been reported [6], lateral meniscus repair, posterior cruciate ligament reconstruction, and anterior to posterior proximal tibial screw fixation [1]. Preoperative identification and accurate reporting of this anatomical variant may enhance surgical awareness and procedural planning [1, 5, 7].

Magnetic resonance imaging (MRI) plays a pivotal role in evaluating knee anatomy, with AATA being most easily detected on axial sequences [1]. Yet, the identification of vascular anomalies relies heavily on radiologists’ interpretation and detection, which may be underdiagnosed, even by experienced radiologists, due to their rarity and subtle imaging appearance, highlighting a diagnostic gap that may benefit from automated detection tools. The integration of artificial intelligence (AI), particularly deep learning techniques, into musculoskeletal imaging has demonstrated promising results in automating diagnostic processes and enhancing detection accuracy [8]. Convolutional neural networks (CNNs) have been widely utilized in medical imaging tasks, providing reliable segmentation and classification of anatomical structures [9].

In this study, we propose the development of a deep learning-based algorithm to automatically detect the presence of an aberrant anterior tibial artery in knee MRI studies. By leveraging a robust dataset and state-of-the-art neural network architectures, our goal is to develop a tool to assist radiologists in the detection of this vascular variant.

## Materials and methods

### Study design

This retrospective study used knee MRI data acquired before the research began. The goal was to develop and validate a classification model distinguishing normal and AATA-present cases, using radiologist annotations as the reference. No direct comparison with other radiologists was performed; instead, we aimed to create a practical tool to support clinical workflows. By automating AATA detection, we seek to enhance knee surgery safety and prevent complications from undetected anomalies.

### Data

A retrospective dataset from the multicenter institution *Diagnóstico da América (DASA)* was acquired after IRB approval, encompassing 2400 knee MRI exams. Of these, 1200 were initially identified through a text-based search of radiology reports containing the term “aberrant anterior tibial artery,” while another 1200 were randomly chosen as controls without mention of this variant. All images were anonymized using the RSNA tool [10].

Five musculoskeletal radiologists (2–7 years of experience), supervised by a senior radiologist (25 years), labeled each MRI slice using the MD.AI Platform [11] (MD.ai, New York), marking the presence (1) or absence (0) of the AATA based solely on image findings. They received about 10 example annotations beforehand. At the end of the labeling process, the same supervising radiologist reviewed all annotations to identify and correct any inconsistencies. No bounding boxes or additional metadata were used.

This dataset was used for training, validation, and internal testing and was restricted to MRI exams performed in 2023. Quality control involved removing duplicate slices, addressing ambiguous cases, and reviewing images with multiple annotations for the same structure. Only T2-weighted and PD axial series were kept for imaging consistency. All MR sequences were performed without IV contrast. An external dataset from the public hospital from *Universidade Federal de São Paulo (UNIFESP)*, a quaternary care hospital, was also obtained under IRB approval. Potential cases were identified by searching for “aberrant” in radiology reports, then labeled and anonymized using the same protocols. Unlike the internal dataset, it was not restricted by acquisition date but followed identical T2-weighted and PD axial filtering.

### Image preprocessing

**Fig. 2:**
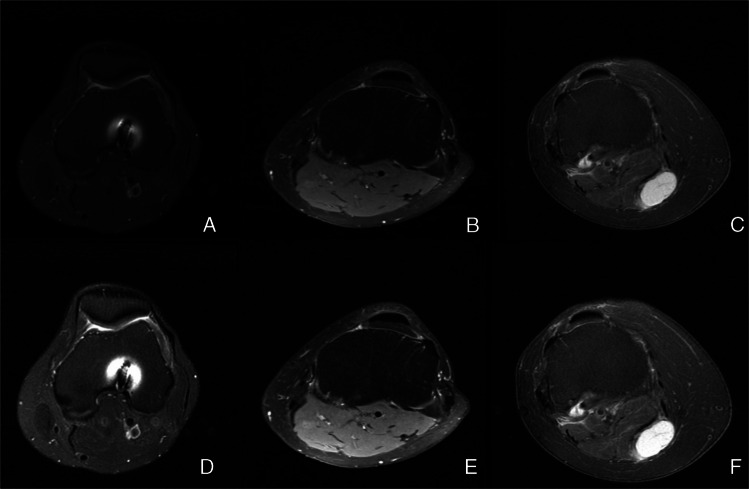
Axial MR images before and after (A/D, B/E, C/F) percentile-based intensity normalization, resulting in more consistent brightness, contrast, and saturation for downstream analysis. After image normalization, the popliteal artery (arrowhead) in figures A/D demonstrates increased conspicuity. Figures A and D demonstrate susceptibility artifacts (straight arrow) related to prior surgical hardware, while figures C and F show a Baker’s cyst (curved arrow). These cases were intentionally included to ensure dataset heterogeneity and to better reflect the variability encountered in knee MRI examinations from the general population

All DICOM files were converted to JPEG Lossless format to facilitate downstream processing. To mitigate the impact of image artifacts, each MRI series was processed in its entirety, and an intensity normalization step was performed by excluding pixel values below the 0.25th percentile (p0.25) and above the 99.75th percentile (p99.75) on a per-series basis. This approach helped to ensure a more uniform and robust representation of brightness, contrast, and saturation across the dataset, thereby improving the overall quality of the input images for subsequent training and analysis (Fig. 2). This preprocessing pipeline was implemented in both internal and external datasets.

### Model development

The internal dataset was split at the patient level into five folds using StratifiedGroupKFold, preventing data leakage and preserving balance between AATA-positive and negative studies. Approximately 60% of the data was allocated for training, 20% for validation, and 20% for internal testing. One fold was held out as the test set, while the remaining four were split into training and validation subsets, without rotating cross-validation.

To enhance robustness, data augmentation was applied to the training set. Small perturbations were introduced via ColorJitter (brightness, contrast = 0.2), RandomHorizontalFlip, and RandomRotation (± 30°), simulating variations in patient positioning. Images were resized to 224 × 224 pixels and normalized using the training set’s mean and standard deviation. Although originally in grayscale, each image was replicated across three channels (3 × 224 × 224) for compatibility with the pretrained model.

A custom CNN was developed with Python 3.8.10 and PyTorch 2.0.1, based on a ResNet10T [12] model from the timm library, pretrained on a large-scale image dataset. A two-dimensional architecture was chosen to prioritize methodological simplicity and scalability. Residual blocks with batch normalization and ReLU activations culminated in global average pooling. The pretrained feature extractor was kept up to its penultimate layer, followed by a custom head consisting of two Dropout layers (*p* = 0.3), a fully connected layer reducing 512 features to 8 with ReLU, and a final linear output producing a single logit. This architecture contains ~ 5.44 million trainable parameters.

Training used a batch size of 128 and binary cross-entropy loss with logits. Adam was employed (learning rate = 0.001, weight decay = 1 × 10^−4^, L1 penalty = 1 × 10^−5^), and an exponential scheduler (gamma = 0.9) reduced the learning rate each epoch for stability.

### Evaluation and statistical analysis

To evaluate the model, slice-level predictions were first generated, with each MRI slice receiving a probability indicating the likelihood of containing the vascular anomaly. These per-slice probabilities were then aggregated to the study level by computing the mean probability for all slices belonging to a given MRI study. A suitable probability threshold was determined using the validation set, and this threshold was chosen to maximize the F1-score, which balances precision and recall, reflecting the clinical trade-off between false positives and false negatives. The same threshold was subsequently applied to internal and external test sets.

Several metrics were used to quantify performance at the patient level, including the binary cross-entropy loss, F1-score, precision, recall, and area under the receiver operating characteristic curve (AUC-ROC). While the slice-level analysis provided insights into how well individual images were classified, the study-level evaluation was more clinically meaningful, as it reflected the final diagnostic determination for each patient’s entire MRI examination.

### Sanity check

To evaluate the model’s interpretability and gain insight into how the trained CNN identified the AATA, a Gradient-weighted Class Activation Mapping (GradCAM) analysis was conducted on a subset of the internal test set. Specifically, five AATA-positive cases that were correctly classified and five AATA-negative cases that were misclassified were selected for closer inspection. GradCAM was applied at the final convolutional layer of the model, capturing the spatial locations that contributed most strongly to the network’s decision.

These GradCAM heatmaps allowed visual verification of whether the network was focusing on anatomically meaningful areas consistent with the presence (or absence) of the tibial artery variant [13].

## Results

### Internal dataset

**Fig. 3:**
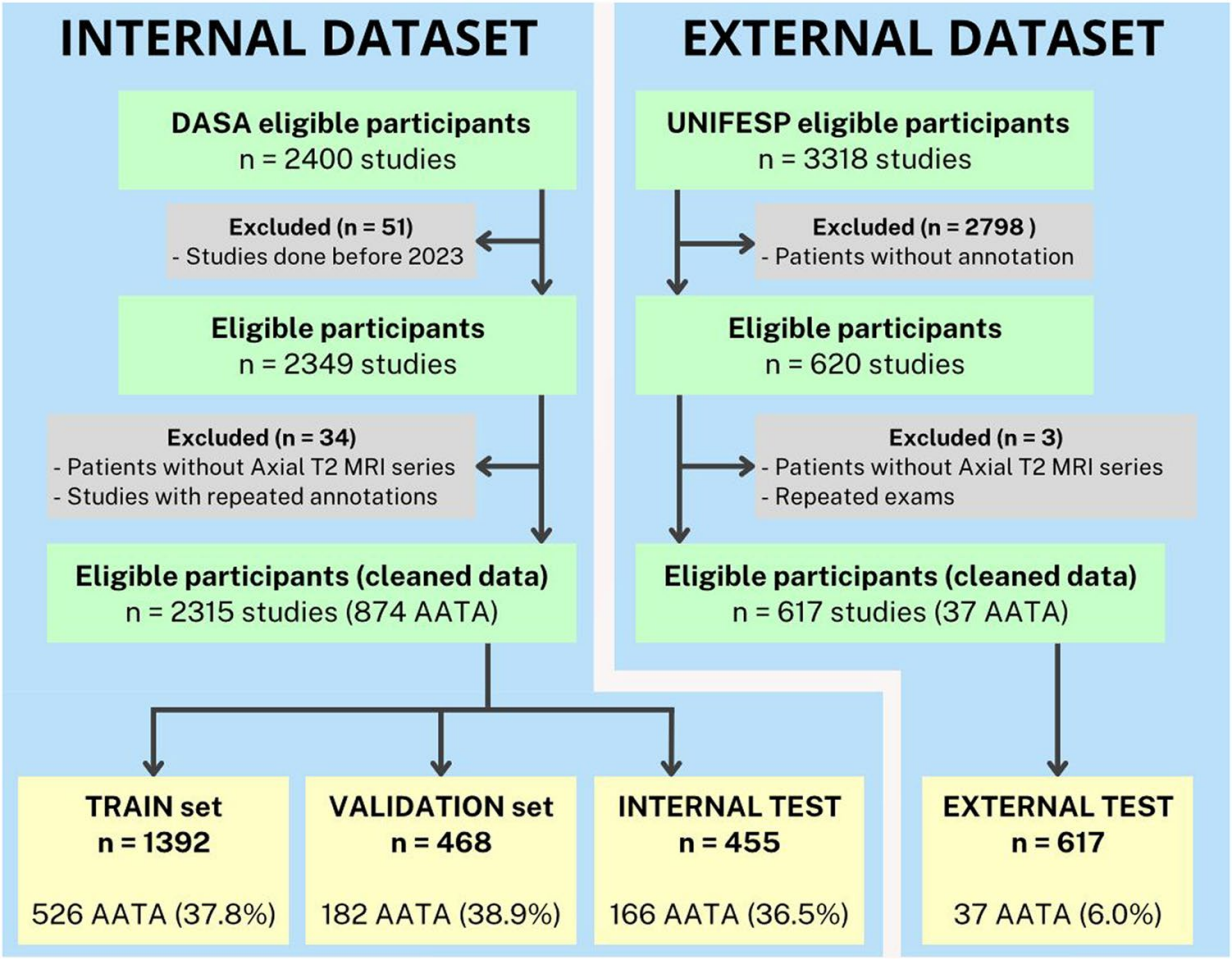
Study selection flowchart. Visualization of the selection process for the internal and external datasets, detailing inclusion and exclusion criteria and the final distribution of knee MRI studies across training, validation, internal test, and external test sets

After removing duplicate exams and correcting misclassifications, approximately 241 studies initially flagged as AATA based on report text search but subsequently reclassified as normal following detailed radiologist image review. The final internal dataset included 1,441 normal studies and 874 with the vascular anomaly, totaling 70,260 knee MRI images across 2315 studies (Fig. 3).

The demographic characteristics of the internal set are summarized in Table 1. The slice thickness had a median of 3.5 mm (range, 2.0–4.5 mm). Other MRI parameters from internal and external datasets are detailed in Supplementary Table 1.

**Table 1:** Demographic and clinical profile of dataset subsets. Demographic and clinical characteristics of the training, validation, internal test, and external test sets

Demographic and clinical profile of dataset subsets				
	Train	Validation	Internal test	External test
AATA—*n* (%)	526 (37.8)	182 (38.9)	166 (36.5)	37 (6.0)
Non-AATA	866	286	289	581
Total patients	1392	468	455	618
Gender—% female	60.1	63.2	55.8	59.8
Age (y)—mean (std)	42.4 (14.7)	42.8 (15.4)	42.2 (14.8)	45.0 (16.0)
Age (y)—max	90	90	90	88
Age (y)—min	5	10	10	6

Guided by the objective of maximizing the F1-score at the patient level on the validation set, a threshold of 0.1717 was established for classifying studies as AATA-positive or normal. Table 2 summarizes the model’s performance across the training, validation, and internal test sets. High F1 and ROC-AUC scores in the validation set reflect strong predictive capability, while applying the same threshold to the internal test set maintained robust performance across accuracy, specificity, recall, precision, negative predictive value, and positive and negative likelihood ratios.

**Table 2:** Model performance metrics across validation, internal test, and external test sets

Model performance metrics				
	Train	Validation	Internal test	External test
F1-score	0.994 (0.989–0.998)	0.963 (0.942–0.982)	0.978 (0.965–0.994)	0.786 (0.674–0.869)
ROC-AUC	0.995 (0.991–0.999)	0.968 (0.950–0.984)	0.982 (0.970–0.995)	0.971 (0.937–0.990)
Accuracy	0.995 (0.992–0.999)	0.972 (0.957–0.985)	0.984 (0.976–0.996)	0.969 (0.955–0.982)
Specificity	0.997 (0.994–1.000)	0.986 (0.972–0.997)	0.989 (0.982–1.000)	0.969 (0.953–0.983)
Recall (sensitivity)	0.992 (0.984–0.998)	0.951 (0.918–0.979)	0.975 (0.950–0.994)	0.972 (0.906–1.000)
Precision (PPV)	0.996 (0.990–1.000)	0.977 (0.954–0.995)	0.981 (0.968–1.000)	0.660 (0.517–0.783)
NPV	0.995 (0.991–0.999)	0.969 (0.949–0.987)	0.986 (0.971–0.997)	0.998 (0.995–1.000)
LR+	429.70% (0.00–882.74)	67.96% (29.62–275.43)	94.01% (0.00–294.20)	31.38% (20.84–54.79)
LR−	0.01% (0.00–0.02)	0.05% (0.02–0.08)	0.02% (0.01–0.05)	0.03% (0.00–0.10)

Each value is reported with its corresponding 95% confidence interval in parentheses

*ROC-AUC* area under the ROC curve, *PPV* positive predictive value, *NPV* negative predictive value, *LR+* positive likelihood ratio, *LR−* negative likelihood ratio

**Fig. 4:**
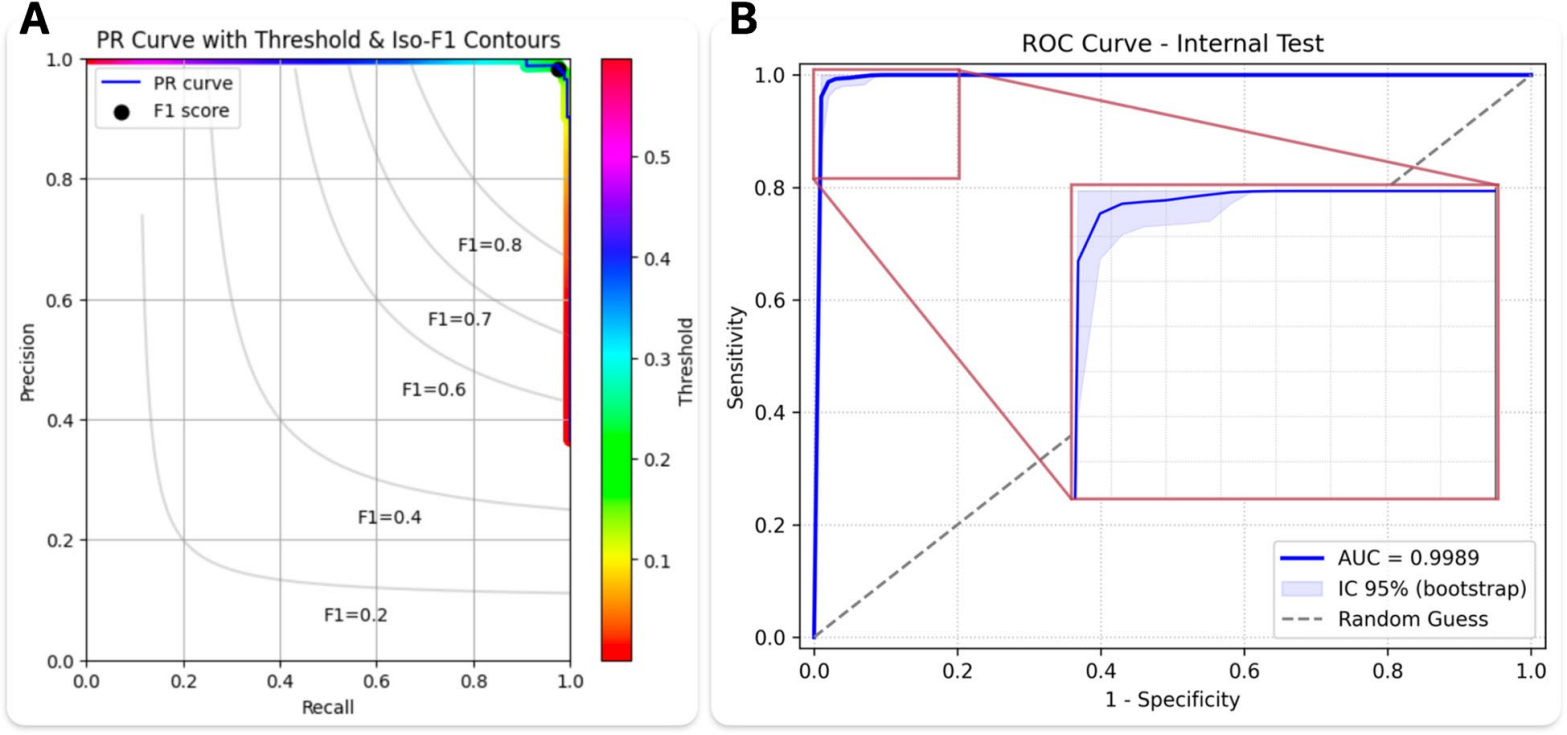
Internal test performance. **A** Precision-Recall (PR) curve with iso-F1 contours and a threshold heatmap, with the black marker indicating the F1-score at the fixed threshold of 0.1717. **B** Receiver operating characteristic (ROC) curve of the model’s performance on the internal test set, with a 95% bootstrap confidence interval shown in light blue. The inset zooms in on the near-perfect region of the curve in the upper-left corner. The dashed diagonal line represents a random-guess baseline

Further analysis using a Precision-Recall (PR) curve with iso-F1 contours (Fig. 4A) and a histogram of predicted probabilities (Supplemental Fig. 1) offered additional insights into model behavior. The PR curve illustrates how precision and recall vary at different thresholds, with iso-F1 lines denoting equivalent harmonic balances. Under the chosen threshold, the model consistently demonstrated high precision and sensitivity. The histogram further confirmed strong discrimination between normal and AATA-positive exams in a moderate-prevalence context. Overall, these findings highlight the clinical applicability of the selected threshold, as only minimal misclassifications were sufficient to reduce precision and F1—yet actual performance displayed minimal degradation. Figure 4B demonstrates the ROC curve for the internal test set.

### External dataset

After preprocessing, the external dataset consisted of 617 studies, performed between 2022 and 2023, with 37 (6.0%) containing the AATA. The demographic characteristics of the external set are summarized in Table 1. The slice thickness had a median of 3.5 mm (range, 2.0–5.5 mm). The markedly lower AATA prevalence in the external dataset (6.0% vs. 37.7% internally) reflects its design to approximate real-world conditions, whereas the internal dataset was intentionally enriched to support model training and validation. This prevalence shift disproportionately affected precision and F1-score in the external test set, while sensitivity and ROC-AUC remained high (Table 2), proving that the model maintained clinically relevant and demonstrating applicability in a quaternary care setting with more comorbid patients.

**Fig. 5:**
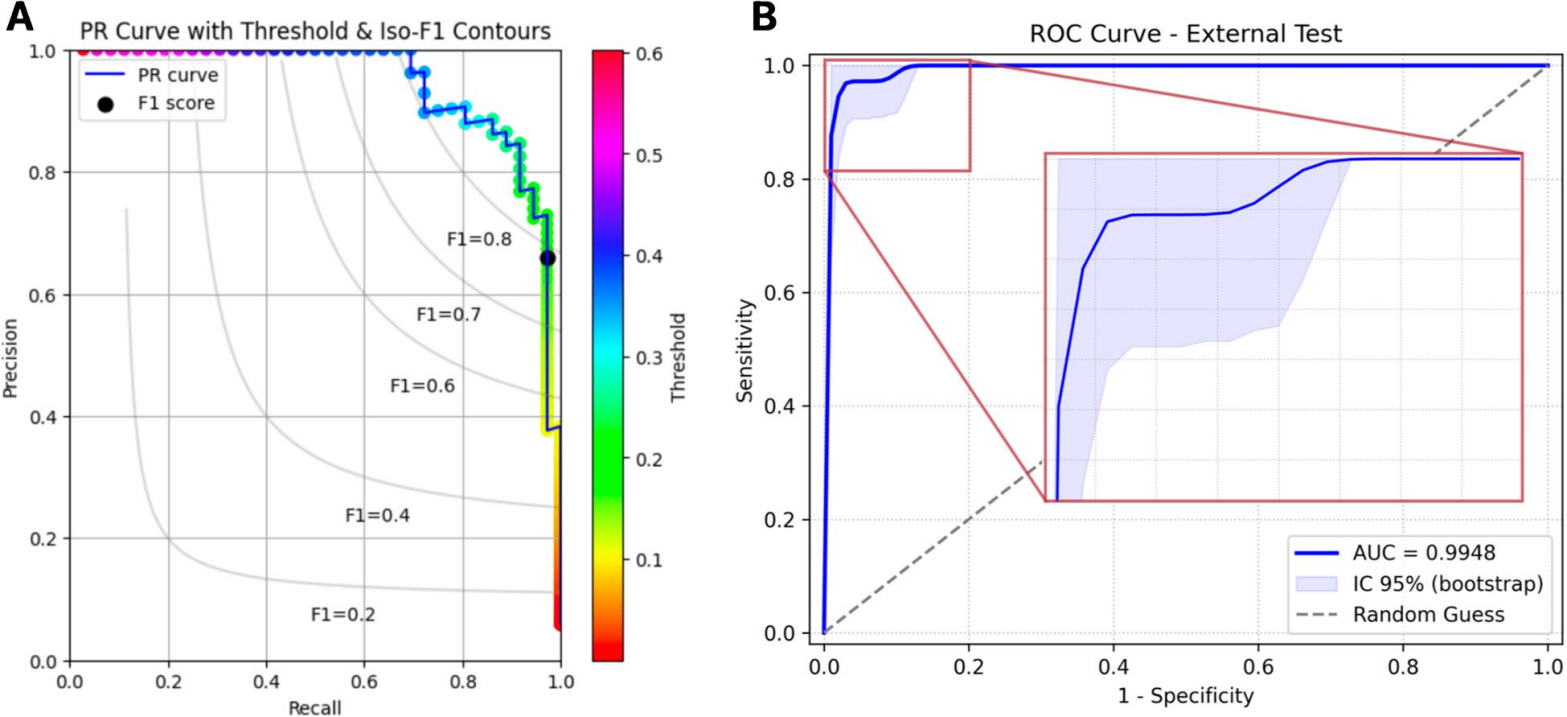
External test performance. **A** Precision-Recall (PR) curve with iso-F1 contours and a threshold heatmap, with the black marker indicating the F1-score at the fixed threshold of 0.1717. **B** Receiver operating characteristic (ROC) curve of the model’s performance on the external test set, with a 95% bootstrap confidence interval shown in light blue. The inset zooms in on the near-perfect region of the curve in the upper-left corner. The dashed diagonal line represents a random-guess baseline

The PR curve (Fig. 5A) and the probability distribution histogram (Supplemental Fig. 2) highlighted the challenge of sustaining high precision under such imbalance. Although accuracy and specificity remained stable, a modest drop in F1 occurred due to a small number of false positives, reflecting a disproportionate impact on precision. Nevertheless, the model effectively distinguished most normal cases from true positives, emphasizing its utility when minimizing false negatives is paramount. Figure 5B demonstrates the ROC curve for the external test set.

### GradCAM analysis

**Fig. 6:**
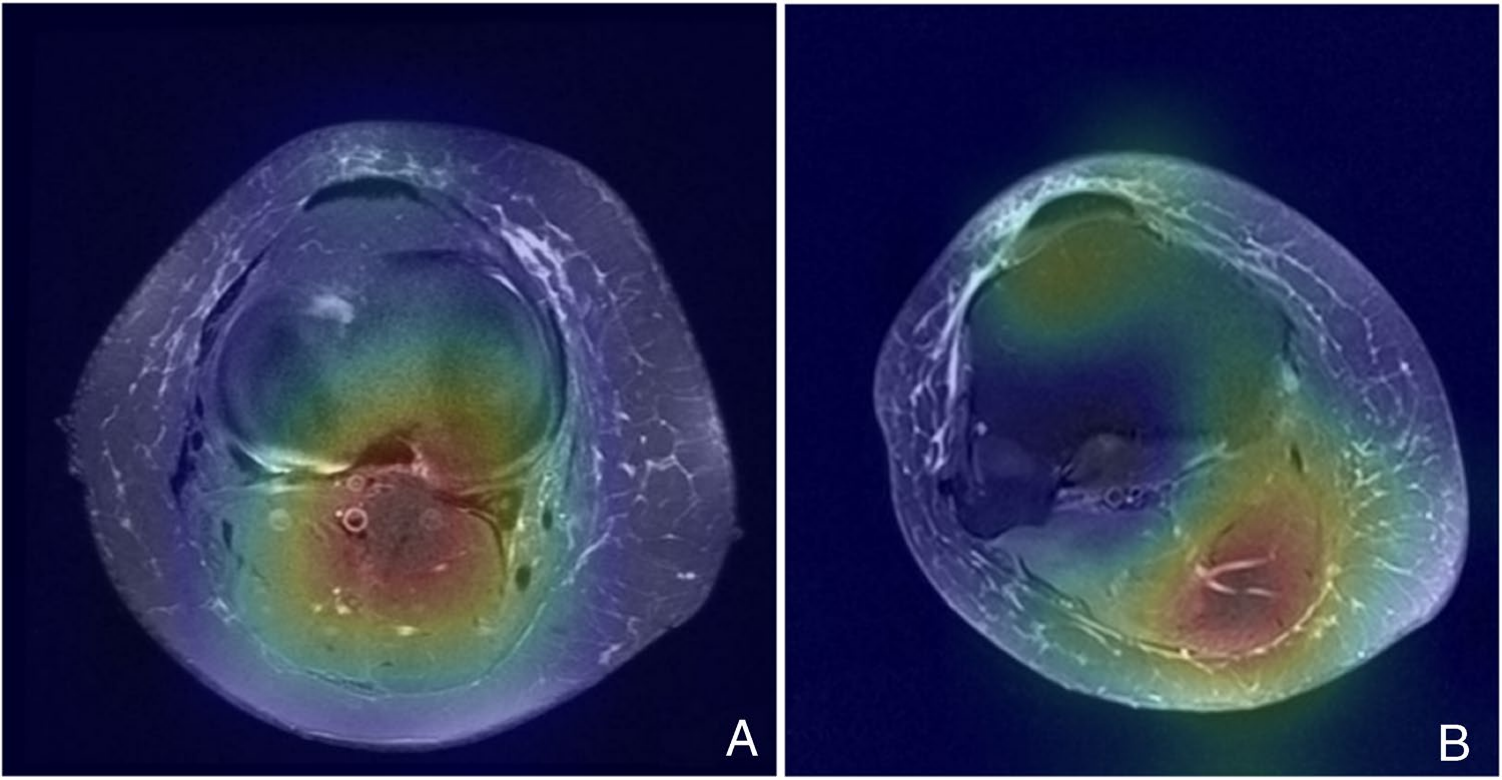
GradCAM heatmap comparison.**A** Presents the vascular anomaly, which was correctly classified with a 100% predicted probability. **B** Absence of AATA, which was misclassified at a 68% predicted probability, illustrating a false positive result

Figure 6 shows example GradCAM heatmaps for both correctly and incorrectly classified studies. In the correctly classified AATA-positive cases, the heatmaps aligned predominantly with the region containing the anomalous artery, indicating that the model was leveraging relevant vascular structures to inform its decision. In contrast, the misclassified example displayed strong activation in the posterior region, likely reflecting a normal vascular structure that the model mistook for the aberrant tibial artery, resulting in a false positive classification. These findings illustrate how anatomically similar vessels can lead to confusion, underscoring the need for more contextual information or a three-dimensional analysis to further refine model performance.

### Analysis of incorrect results

A confusion matrix generated on the external test set showed 536 true negatives, 35 true positives, 1 false negative, and 18 false positives. Most misclassifications involved atypical anatomical or imaging artifacts. For instance, the model erroneously identified a clinically insignificant proximal bifurcation as the AATA. These findings highlight the model’s tendency to overcall certain vascular anomalies, particularly when encountering unusual anatomical or acquisition scenarios.

## Discussion

To date, no study has attempted to detect the AATA variant using artificial intelligence algorithms. Although rare [1, 3], the AATA is encountered in routine knee MRI, one of the most frequently performed musculoskeletal examinations. Despite its potential clinical relevance related to the risk of iatrogenic injury, which may result in worse clinical outcomes and surgical complications if unrecognized, this vascular variant may be overlooked or underreported, as it is uncommon and not routinely emphasized during standard knee MRI interpretation. Automated detection tools may serve as an adjunct to the reporting radiologist by drawing attention to such findings. This perspective provides the rationale for exploring a deep learning–based approach to improve detection consistency.

In this study, we adopted a 2D slice-by-slice classification approach to detect the presence or absence of an aberrant anterior tibial artery in knee MRI scans. This strategy contrasts with most existing studies [14, 15], which primarily rely on segmentation techniques to evaluate vascular structures. For instance, U-Net architectures have been widely used for vessel segmentation in medical imaging due to their ability to capture both contextual and fine anatomical details. Additionally, 3D convolutional neural networks have demonstrated effectiveness in segmenting brain, retinal, and thoracic vessels, highlighting the advantages of three-dimensional approaches for vascular analysis [14, 15], although they require higher computational complexity and more homogeneous acquisition protocols, which could introduce unnecessary complexity for the aims of this study.

This study demonstrates the feasibility of using deep learning approaches to detect vascular anomalies in MRI exams, as we successfully developed a neural network for the detection of AATA in knee MRI studies, achieving a high F1-score. The strong performance of our model in internal validation demonstrates its robustness in identifying vascular anomalies with high accuracy.

Furthermore, the external test set also yielded a favorable F1-score, but with a marked drop in precision (0.66 vs. 0.98 in the internal test set), despite maintaining high recall (0.972) and accuracy (0.969). This discrepancy can be partly explained by the much lower prevalence of AATA in the external cohort (6% vs. 36.5% internally). In addition, intrinsic differences between populations likely contributed, as the training data were obtained from an outpatient-based multicenter institution, whereas the external dataset came from a quaternary care hospital with patients presenting more severe comorbidities and more complex imaging findings. Technical factors such as older MRI scanners and higher patient throughput may also have reduced image quality.

Despite these promising results, several limitations warrant attention. First, the training data were exclusively obtained from a private multicenter institution, which may not fully reflect the spectrum of imaging protocols, patient demographics, and disease presentations encountered in broader clinical practice. Second, our model processes each slice as a discrete 2D image, which may not capture contextual cues from adjacent slices. This approach could potentially overlook subtler anatomical indicators or spatial relationships relevant to the confident identification of vascular anomalies. Lastly, radiologist consensus on MRI served as the reference standard, as no angiographic or surgical confirmation was available, which may have led to misclassification in borderline cases.

Overall, this study demonstrates that a deep learning model can reliably detect the arterial anomaly on knee MRI, showing high diagnostic performance across both internal and external test sets. These results indicate that automated AATA detection is feasible and may support radiologists by providing consistent recognition of this vascular variant. Although further work is required to address the limitations and evaluate the real impact in the radiologist’s workflow.

## Supplementary Information

Below is the link to the electronic supplementary material.

ESM 1 (DOCX 1.39 MB)

## Data Availability

Data analyzed during the study would not be available due to privacy concerns. The code used in this study is publicly available at https://github.com/cassianopb/aata-detection-mri.
