# Supplementary material for: Deep learning-based identification of aberrant anterior tibial artery on knee MRI: a brazilian multicenter study
Source: Skeletal Radiol. 2026 Mar 23;55(7):1617–25. doi: 10.1007/s00256-026-05198-z (PMC13198507; doi:10.1007/s00256-026-05198-z)
Supplement: Supplementary file 1 — (DOCX 1.39 MB) [file 256_2026_5198_MOESM1_ESM.docx]

**
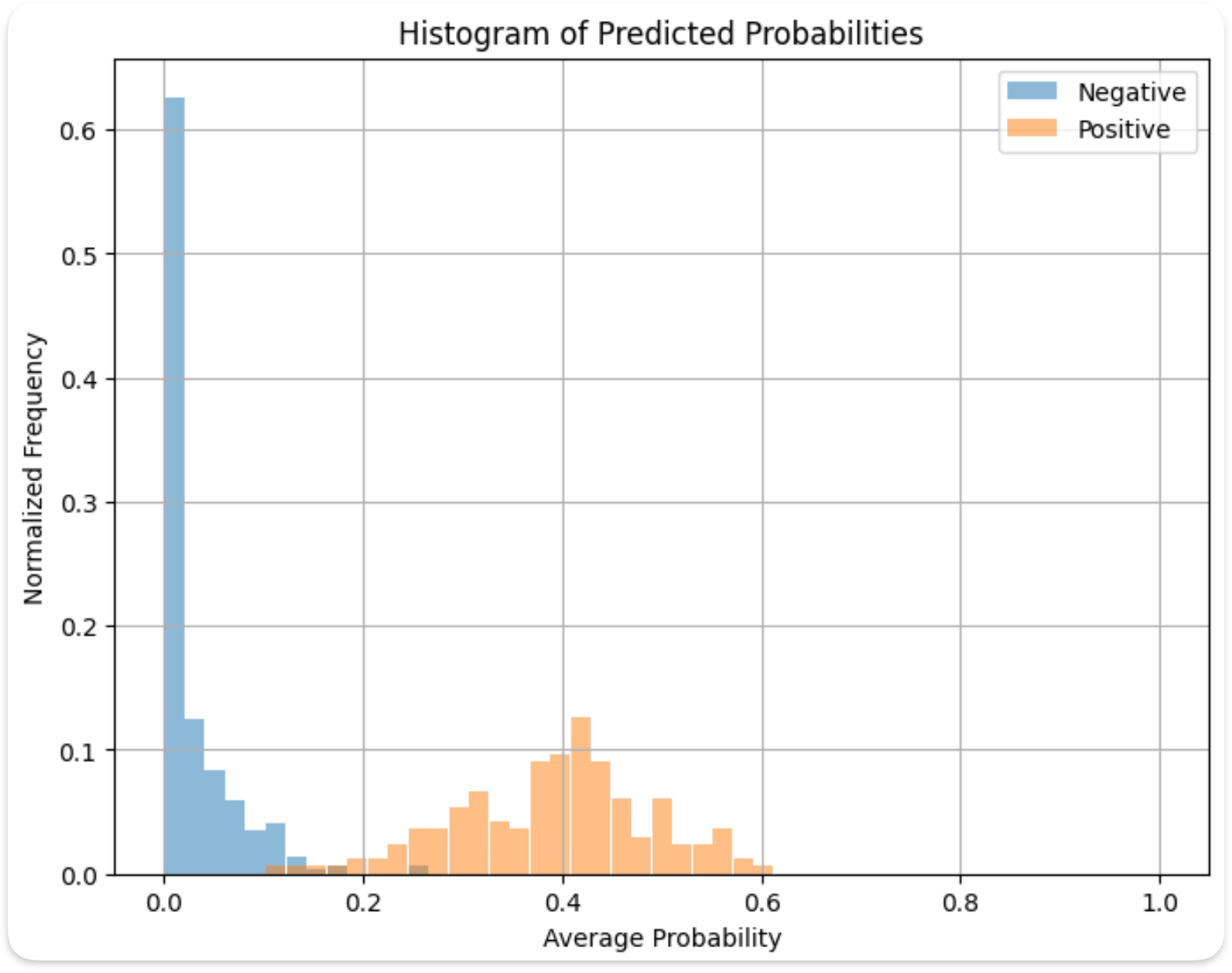
**

**Supplemental Figure 1.** Normalized histogram of predicted probabilities from internal test set for the positive and negative classes, illustrating the distribution of predictions and the impact of class imbalance.


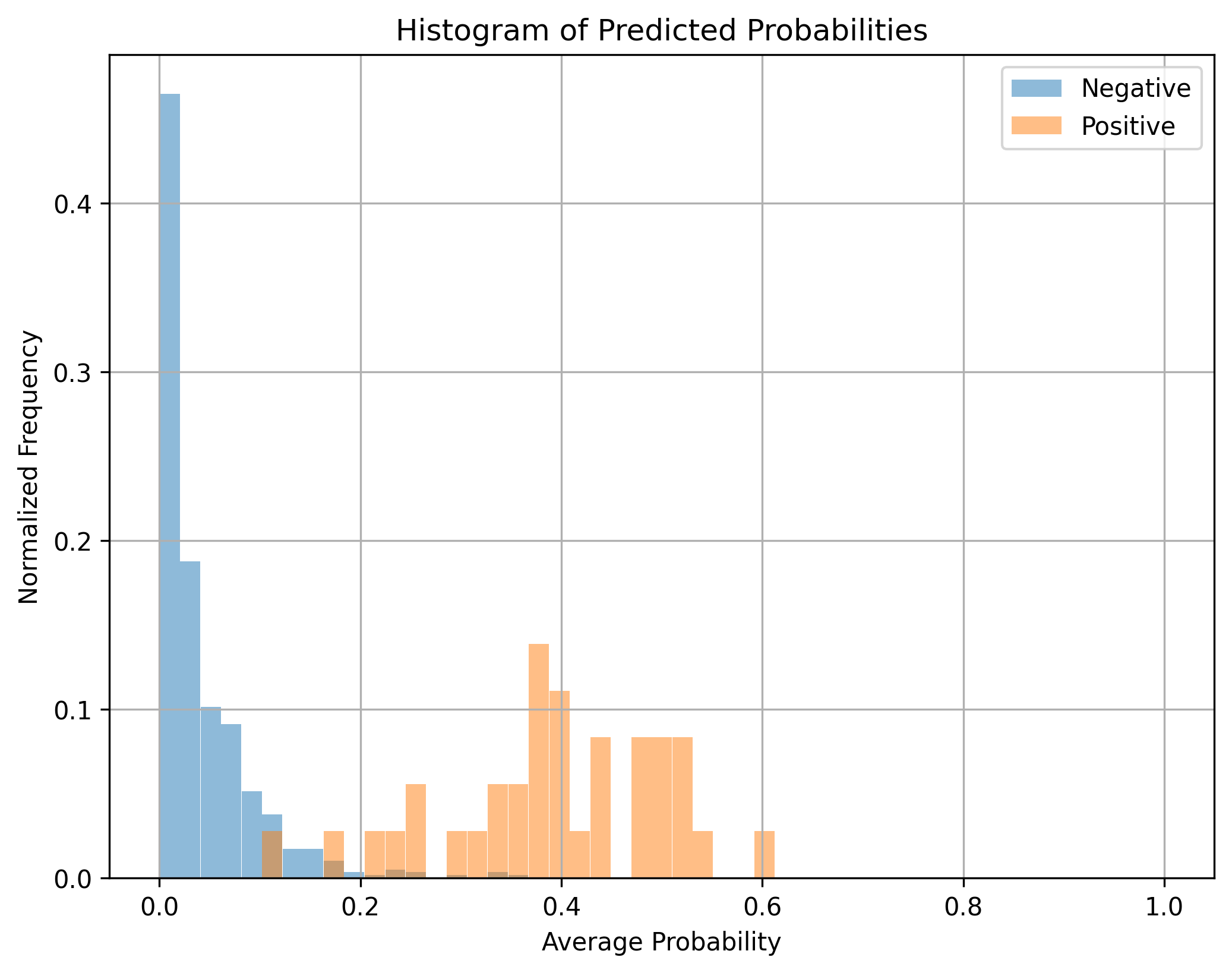


**Supplemental Figure 2.** Normalized histogram of predicted probabilities from external test set for the positive and negative classes, illustrating the distribution of predictions and the impact of class imbalance.

| **MRI Parameters of Dataset Subsets** | | | | |
| --- | --- | --- | --- | --- |
| MRI Parameter | Train | Validation | Internal Test | External Test |
| Manufacturer | GE MEDICAL SYSTEMS (887); SIEMENS (365); TOSHIBA (68); Philips Medical Systems (66); Siemens (5); CANON_MEC (1) | GE MEDICAL SYSTEMS (307); SIEMENS (116); TOSHIBA (26); Philips Medical Systems (16); Siemens (3) | GE MEDICAL SYSTEMS (297); SIEMENS (118); TOSHIBA (20); Philips Medical Systems (19); Siemens (1) | Philips Medical Systems (320); SIEMENS (287); GE MEDICAL SYSTEMS (12); TOSHIBA (1) |
| Model Name | Signa HDxt (255); Optima MR360 (184); Brivo MR355 (145); SIGNA Explorer (121); MAGNETOM_ESSENZA (85); Avanto (78); SymphonyTim (77); Vantage Elan (68); Ingenia (55); OPTIMA MR360 (53); Verio (45); Optima MR450w (45); BRIVO MR355 (41); Espree (29); SIGNA EXCITE (20); Sempra (20); Aera (17); SIGNA Creator (13); Achieva dStream (8); Skyra_fit (8); Symphony (6); MAGNETOM Sempra (5); SIGNA Voyager (3); GENESIS_SIGNA (3); Intera (3); SIGNA Pioneer (2); DISCOVERY MR750w (2); Orian (1) | Signa HDxt (81); Optima MR360 (70); SIGNA Explorer (42); Brivo MR355 (40); MAGNETOM_ESSENZA (30); Vantage Elan (26); SymphonyTim (26); Avanto (24); OPTIMA MR360 (24); BRIVO MR355 (16); Optima MR450w (15); Ingenia (13); Espree (9); Verio (8); Sempra (8); SIGNA EXCITE (6); Aera (6); SIGNA Creator (6); MAGNETOM Sempra (3); Symphony (3); DISCOVERY MR750w (3); SIGNA Voyager (2); Achieva dStream (2); Skyra_fit (2); SIGNA Pioneer (1); Intera (1); GENESIS_SIGNA (1) | Signa HDxt (87); Optima MR360 (69); Brivo MR355 (42); SIGNA Explorer (39); Avanto (25); MAGNETOM_ESSENZA (24); OPTIMA MR360 (21); Vantage Elan (20); SymphonyTim (19); Ingenia (16); Espree (14); Aera (13); Optima MR450w (12); BRIVO MR355 (11); Verio (8); Sempra (8); SIGNA EXCITE (7); Skyra_fit (5); SIGNA Creator (4); Achieva dStream (3); SIGNA Pioneer (3); Symphony (2); GENESIS_SIGNA (1); DISCOVERY MR750w (1); MAGNETOM Sempra (1) | Achieva (317); Skyra (277); SIGNA Explorer (5); Aera (4); Ingenia (3); Signa HDxt (3); Optima MR360 (2); MAGNETOM_ESSENZA (2); Brivo MR355 (2); SymphonyTim (1); Avanto (1); Sempra (1); Vantage Elan (1); Verio (1) |
| Field Strength (T) | 1.5 (1333); 3.0 (57); 0.2 (2) | 1.5 (454); 3.0 (14); | 1.5 (437); 3.0 (17); 0.2 (1) | 3.0 (595); 1.5 (25) |

**Supplementary Table 1:** *Description of the MRI parameters for Internal and External Datasets*
